# Concerned and conscious, but defenceless - the intersection of gender and generation in child malnutrition in Indonesia: a qualitative grounded theory study

**DOI:** 10.1080/16549716.2020.1744214

**Published:** 2020-05-06

**Authors:** Masoud Vaezghasemi, Ann Öhman, Nawi Ng, Mohammad Hakimi, Malin Eriksson

**Affiliations:** a Department of Epidemiology and Global Health, Umeå University, Umeå, Sweden; b Umeå Centre for Gender Studies, Umeå University, Umeå, Sweden; c Centre for Demographic and Ageing Research, Umeå University, Umeå, Sweden; d Centre for Health and Nutrition Research Laboratory, Faculty of Medicine, Gadjah Mada University, Yogyakarta, Indonesia; e Department of Health Behaviour, Environment and Social Medicine, Faculty of Medicine, Gadjah Mada University, Yogyakarta, Indonesia; f Department of Social Work, Umeå University, Umeå, Sweden

**Keywords:** Double burden of malnutrition, nutrition transition, gender, intersectionality, grounded theory, qualitative study, focus group discussions, Indonesia

## Abstract

**Background**: Several studies in Indonesia have shown the protective effect of women-headed households on the double burden of malnutrition (coexistence of undernutrition and overnutrition in a household). Many other studies have presented a positive impact on children’s health and conditions when women are educated, have higher social capital and have control of income and its intra-household allocation. However, how women’s status affects the nutritional status of a household and, in particular, of children still remains understudied.

**Objective**: In this study, our aim was to explore the role of gender relations and contextual factors for overnutrition and undernutrition among children within a household.

**Method**: We conducted a qualitative study in two provinces of Indonesia: Central Java (urban and rural) and Jakarta (central and suburban) among 123 community members (59 men and 64 women). We utilised principles of constructivist grounded theory in conducting this study, and focus group discussions were chosen as a tool to collect data.

**Results**: Three categories were constructed, capturing the significance of: (i) the man is dominant within the family (gendered power relations), (ii) the environment that makes the unhealthy choice the easy choice (the emerging obesogenic environment) and (iii) parents’ being concerned but unable to control their children’s eating habits (intersection of gender and generational relations) in child malnutrition.

**Conclusion**: Community health and nutrition programmes should help both women and men within the context of households to acknowledge and respect women’s status. More importantly, these programmes should involve men when it comes to children’s nutritional habits and consider them as an important factor in the realisation of gender equality and empowerment. Furthermore, it is increasingly important to recognise the implication of the availability and accessibility of junk food among children.

## Background

An increasing number of Low and Middle-Income Countries (LMICs) must shoulder a ‘double burden’ of malnutrition, which is the persistence of undernutrition (i.e. underweight, stunting and wasting, especially among children) along with a rapid rise in overweight, obesity (especially among women) and diet-related chronic diseases [1–3]. The global burden of child undernutrition has significantly lowered since the introduction of the Millennium Development Goals (MDGs) in 1990. Notwithstanding global and regional improvements, however, a high proportion of undernourished children still live in Africa and parts of Asia [4].

In Indonesia, while the prevalence of overweight and obesity is increasing [5,6], undernutrition remains a public health concern [7], especially among children. Moreover, t was reported that stunting coexists with overweight among children under five years of age and that stunted children are more likely to become overweight later in life [8]. The protective effect of women-headed households on the double burden of malnutrition in Indonesia has been shown in several studies [9,10]. Many other studies in Indonesia and elsewhere have presented a positive influence on children’s health and conditions when women are educated [11], have social capital [12] and have control of income [13,14] and its intra-household allocation [15].

With regard to child malnutrition, previous studies have mainly focused on the maternal perspective by overemphasising interventions concerned with breastfeeding, micronutrient nutrition, complementary and therapeutic supplementary feeding. There is no doubt about the effectiveness of these approaches; however, merely focusing on the maternal perspective will overlook the broader context of the household social and economic susceptibilities to which both the child and mother are exposed [16]. Studying child malnutrition in India, Corsi et al. showed a higher population attributable risk following the inclusion of parental characteristics, such as parents’ stature, Body Mass Index (BMI) and, above all, household wealth. Accordingly, they suggest the need to move ‘beyond the maternal perspective towards a household lens’ [16]. In fact, the importance of the household as a social context has also been raised in regards to the variation of BMI in Indonesia [17]. In addition, Indonesia is undergoing a nutrition transition, as indicated by the increasing prevalence of overweight and obesity. This highlights the importance of contextual factors, i.e. the obesogenic environment, beyond the maternal and households’ characteristics.

A systematic review of women’s empowerment and child malnutrition status in South Asia [18] reported that women’s low social status is generally associated with children’s poor nutritional status; however, there is a great deal of disparity in the literature and a need for further studies. Moreover, a systematic review on child health inequalities in Indonesia found that the association between mothers’ household decision-making and child health is rather unexplored [19]. Nevertheless, quantitative approaches to analysing data, which are generally the only available sources for population studies, would most often miss the relational and emergent mechanisms within households and between their individuals who encounter different forms of malnutrition; for instance, undernutrition among children and overnutrition among mothers.

The general ideology on gender is reflected in Indonesia’s national organisation called *Dharma Wanita* (Women’s Dedication). After being officially regulated in Marriage Law No. 1/1974 (State Secretariat of the Republic of Indonesia, 1974), the law states that, although husbands and wives have equal rights within their marriage, the husband is the head of the household – a fact which is stated on family cards – and that the main role of women involves being their husband’s companion and caregiver to their children. This gendered social structure of domination exists in patriarchal societies such as Indonesia where men, who are considered to be the main breadwinners for the family, are given the power and right to control women [20]. On the contrary, women are expected to be the household caretakers, show obedience to their husbands and be responsible for child rearing [21,22]. These constructs and hegemonic values place men as superiors within the arena of the public sphere, the workplace and the family, so they effectively play a role in maintaining the subordination of women [23]. In many parts of Indonesia, however, a woman’s role is critically important in her households, particularly in managing household finances and generating income through formal employment or informal activities [24]. Such active economic roles inside and outside of the household empower women to control the finances and gives them authority in the family [25,26]. However, how women’s status affects the nutritional status of a household and, in particular, of children, still remains understudied. In this study, our aim was to explore the role of gender relations and contextual factors for overnutrition and undernutrition among children within a household in two provinces in Indonesia – Central Java and Jakarta.

### Theoretical framework

In this study, we applied Connell’s relational theory of gender. This approach considers gender as a multidimensional structure functioning in a complex social context [27]. Connell embraces a social constructionist perspective on gender and defines gender as ‘a pattern of social relations in which the position of women and men are defined, the cultural meaning of being a man and woman are negotiated, and their trajectories throughout life are mapped out’ [28,p.839]. Accordingly, gender comprises not only direct relations between men and women but also indirect relations through work, technology or symbols that are constantly changing and negotiated. Although less often considered in public health literature [29], a relational approach to gender provides enriched understanding of how different circumstances can influence women’s and men’s health and illnesses. Hence, gender relations are defined as the relations of power and dominance that structure the life chances of women and men in specific social contexts [30].

Connell’s theory recognises four dimensions of gender relations, namely 1) power relations and, in particular, men’s domination and power over women; 2) production relations, meaning a gendered division of labour in that certain tasks are mainly done by women and others by men; 3) emotional relations, implying different expectations of men and women to engage and commit in emotional relationships and 4) symbolic relations, dictated by cultural beliefs of how men and women should behave and act [31]. These dimensions are closely linked in practice and may cause different forms and degrees of inequalities for women that vary over time and place. We consider Connell’s relational theory to be a useful tool in describing and analysing how gendered power structures function in a specific context and how the intricate relationships between men and women within a household work with regard to child overnutrition and undernutrition.

## Methods

### Study design and analytical approach

We utilised principles of constructivist grounded theory in conducting the current study [32]. This analytical approach implies ‘systematic yet flexible guidelines for collecting and analysing qualitative data to construct theories grounded in the data themselves’. Thus, the basis for this analysis was our data, to avoid being overly influenced by certain pre-determined theories. Nevertheless, in accordance to the principles of constructivist grounded theory, acknowledging that the result is an interpretation conducted by the researchers, we used gender relations as sensitising concepts to provide a starting point for building a grounded theory analysis. Sensitising concepts provide a general sense of reference and guidance in approaching empirical instances and suggest directions rather than enabling researchers to move directly to the example that is related to a specific theory or idea [33]. Therefore, it gives the researchers a sense of how detected examples of phenomena might fit within conceptual categories. Focus group discussions (FGDs) were chosen as the data collection tool because we wanted to capture a broad range of norms and attitudes among men and women with regard to a focused theme, which, in this study, was overnutrition and undernutrition among children within the household context [33,34].

### Study setting

We conducted FGDs in two provinces of Indonesia: Central Java (urban and rural) and the capital city of Jakarta (central and suburban). These two provinces were chosen based on findings of our previously conducted quantitative study on the double burden of malnutrition in Indonesia, which identified Central Java as one of the two provinces with the lowest prevalence of double burden households and Jakarta as the province with the highest prevalence [10]. In addition, we found a statistically significant association between double burden households and places of residence [10]. Therefore, this qualitative study was conducted in central and suburban areas in Jakarta as well as in urban and rural areas in Central Java.

### Participants and group compositions

The research group identified community leaders and informed them about the purpose of the study and the characteristics of the participants. Community leaders were asked to select married adults living in households with more than two members from different socioeconomic backgrounds and invite them to the study. In total, 123 community members from 16 villages (eight in Central Java and eight in Jakarta) participated in this study (59 men and 64 women). We assumed such social and geographically diverse groups would provide us with a broader understanding of the topic and allow us to explore the different perceptions and attitudes of men and women. Based on our pre-understanding about the context in which the gender composition of focus groups may compromise women’s ability to voice opinions on all issues, especially on matters related to gender, men and women were interviewed separately; hence, due to these practical fieldwork arrangements, a ‘pure’ theoretical sampling in accordance with grounded theory was not possible. Table 1 presents the geographical locations and number of study participants. The participants were married adults, aged 27–67 (except for two adolescents and one divorced woman), mainly Javanese with secondary education. The women were mainly housewives – especially in Jakarta – and the men had private or non-governmental jobs.

**Table 1.** Number of participants and geographical locations in qualitative focus group discussions (FGDs) in Indonesia, 2013

Geographical location	Groups	Informants	Men	Women
Central Jakarta	4	23	11	12
Suburban Jakarta	4	27	12	15
Urban Purworejo	4	34	18	16
Rural Purworejo	4	39	18	21
	**16**	**123**	**59**	**64**

### Data collection

We conducted 16 FGDs separately for men and women during 1 July to 24 August 2013. Eight FGDs were conducted in Central Java (four in urban and four in rural areas) and eight in Jakarta (four in central and four in suburban areas). A focus group consists of a small and relatively homogenous group of participants who are brought together to explore attitudes and perceptions, feeling and ideas about a certain topic [35]. It is not just a haphazard discussion and brainstorming session among people who happen to be available; it is a well-planned endeavour moderated by a trained and skilled interviewer to provide a structure within which participants may interact and express their thoughts and feelings [36].

We intended to form homogenous groups of participants by inviting mainly married adults – men and women – separately. In order to create an open atmosphere for discussion, participants were positioned around a table or in a circle on the floor. They were then asked to talk one by one and give others a chance to share their stories. Those who intended to dominate the discussion were gently and politely asked to stop and told ‘thank you for sharing your story with us; maybe it is time to hear from others’. We assured participants that there is no right or wrong way to talk and asked them to freely express their ideas in favour or against the opinions of others. Each FGD lasted one to two-and-a-half hours. They were voice recorded, transcribed verbatim in Bahasa Indonesia and finally translated into English.

Most of the FGDs were performed in community halls but some (in Jakarta) were performed in community leaders’ or participants’ homes. MV, the researcher, together with the local assistant conducted all the FGDs. Due to the language barrier, MV acted as the moderator’s assistant, and the local assistant moderated the discussions. The local assistant was Javanese and fully familiar with English, Indonesian (Bahasa Indonesia) and also Javanese. In addition, she was experienced in conducting qualitative interviews and gender-related studies in several international projects in Indonesia. Throughout several meetings and discussions, she became well informed about the objectives and scope of the study, and there were constant discussions between her and MV before, within (when necessary) and after each FGD. Apart from being aware of the topic being discussed, it was vital that the moderator was culturally and socially familiar with the participants as well as the context. This enabled her to act as a cultural interpreter, not just a linguistic translator.

We used open-ended and non-judgmental questions to facilitate a detailed discussion of the topic [32,35]. We developed three sets of questions to encourage informants in an open exchange and discussion guided by these questions: (i) introductory or engaging questions (for instance, ‘What do people usually eat in this area?’ or ‘Why, in some households, are there both underweight and overweight individuals?’); (ii) exploratory or linking questions (i.e. ‘What does it mean to be the head of a household?’ and ‘how do relations between men and women within the household affect the household’s health and nutrition?’) and (iii) closing questions (i.e. ‘Is there something else in relation to this that you would like to talk about?’) Furthermore, in order to stimulate a discussion around informants’ values and attitudes, we exhibited pictures and figures presenting overweight and underweight people that may sometimes happen to be in the same household. We used these as a starter at the beginning of the FGD in order to provide enough context and information for informants to have an understanding of issue being discussed [37,38]. Ultimately, the question in focus was how gender relations influence the double burden of malnutrition within a household.

### Steps of the analysis

We analysed data on group level and in multiple phases. During the data collection, some FGDs were transcribed and translated by the local assistant and reviewed by MV. After each FGD, MV and the local assistant discussed the process, and necessary changes and preparations were carried out for the next FGDs. Initial notes or memos were subsequently discussed with MH and NN for further investigations.

After data collection, the analysis continued following the steps suggested by Charmaz [39], i.e. open/initial coding, focused/selective coding and theoretical coding. MV, AÖ, ME and NN read the transcripts and made initial rounds of line-by-line open coding, which was conducted on a subset of FGDs by each member of the research team separately. Transformation of data into codes was done by a close reading of the transcriptions. At this stage, we tried to be as close to the text as possible rather than applying pre-existing theories to the data. *In vivo* codes were identified and used to indicate participants’ own views and actions in the coding itself. We reviewed and compared these codes in order to include different perspectives and attain a negotiated outcome. Focused or selective coding was then done by selecting and clustering the most significant and frequent initial codes as well as coding the initial codes with a more specific focus to examine and analyse large batches of data [35,39]. We then selected clusters of codes and re-clustered them into new clusters and preliminary categories. In this step, in line with the sensitising concept, only the clusters of codes that we interpreted as most relevant or central to fulfilling the aim of the study were selected. Finally, theoretical coding was performed to conceptualise how the substantive codes could relate to each other as hypotheses to be integrated into a model [32]. Theoretical coding helped to specify the possible relationships between categories developed through focused coding. These codes are meant to be integrative and help to tell a coherent, analytical story [39]. Table 2 illustrates an audit trail of moving from text to category in the analytical process. The programme NVivo (Version 11.1.1 (1551)) was used to analyse the data. Note: this study is part of a PhD dissertation; thus, some of the quotes presented in the results section have already been published [40].

**Table 2.** Illustrating an audit trail of moving from text to category in the analysing process

Text	Open codes	Clusters	Category
*From Javanese culture, from our religion, which states that the male is an imam (leader)*. *A male is a leader; we, as women, are companions. If something is wrong, we remind him, we talk to him. Still it is the man who leads.*	Household head is a man Culture and religion consider men as leaders Men lead the household Women are companions for men	Being the head of a household implies being a man	**The man is dominant within the family and influences family eating behaviour**
*The male makes a living, educates the wife and children, keeps the family’s dignity, and communicates and socialises with neighbours and relatives. A wife is also a household leader. She keeps her husband’s wealth. If a husband is not at home, she can be his representative. However, the full responsibilities are the husband’s. Yet a wife also plays some roles. She is not a household leader, but she is great. A wife’s support is amazing.*	Men should make a living Men should educate their wife and children Men should keep the family’s dignity Men should communicate and socialise with neighbours and relatives A wife’s role is to keep their husband’s wealth Wives’ support is amazing	Men being leaders and women being companions for men	

## Results

The analyses resulted in three categories conceptualising how gender relations and social contextual factors influence overnutrition and undernutrition within a household, namely (i) ‘the man is dominant within the family’, which represents relations between husband and wife in the household; (ii) ‘the environment makes the unhealthy choice the easy choice’, which represents the view of an existing and dominant food market that is not easy to oppose and (iii) ‘parents are concerned but not able to control children’s eating habits’, which represents power relations between mothers, fathers and their children. We argue that the three categories represent i) gendered power relations, ii) an emerging obesogenic environment and iii) intersections of gender and generation. In the following sections, we present the three categories.

### ‘The man is dominant within the family and influences family eating behaviour’

This category focuses on socially and culturally constructed and institutionalised gendered power relations and how society and culture influence the power arrangement within a household. It reflects the gender order within households among children, men and women and also when it comes to the way gender influences children’s eating behaviour.

It is evident from our data that women are at the bottom of the family hierarchy. According to the informants, such power relations and gender arrangements are rooted in the Javanese culture as well as in the Islamic religion.

*‘ … from Javanese culture, from our religion, stating that the male is an imam (leader).’* Participant in women group in rural Purworejo (FGD 3).

Men are leaders because ‘it is destiny and nature. Women are below men.’

Participant in women group in Suburb Jakarta (FGD 16).

*‘A male is a leader; we as women group are companions. If something is wrong, we remind him, we talk to him. Still, it is the man who leads.’* Participant in woman in Central Jakarta (FGD 15).

In addition to this, women consider this to be a way of expressing their respect towards men, by putting them at the top of the family hierarchy. Mothers ‘naturally’ put themselves after the family and take the burden of leaner times by eating last. Children are considered to be a special gift from God and are highly privileged and favoured among Indonesian families; when it comes to food, they are always first, especially when food is limited. Children are the priority in receiving food first because they are in a period of growth and need to develop a healthy condition. Based on the information reported in all of the FGDs, both boys and girls within families receive equal food and treatment, and no preferences are considered. Nonetheless, when children grow up, the priority goes to fathers.

*‘In my family, we put our children first because they are still young. When they are grown up, perhaps the priority will turn towards the father. It does not go to the mother because, generally, in our traditional community, a wife will put her husband first. They take the second turn.’* Participant in men group in rural Purworejo (FGD 1).

Despite the presence of a hegemonic – nationally, religiously and culturally institutionalised – gender ideology, the participants’ responses showed signs of changes occurring in gender relations. While the majority of men and women still posit a strong sense of compliance with the institutionalised beliefs and values in gender relations, there have been some indications and manifestations of transitions occurring in the practices or roles of gender within the context of the household. For example, doing household chores such as cooking and cleaning together during weekends and sharing ideas to make decisions were also mentioned by some of the informants in the FGDs – for instance, in urban Purworejo and suburban Jakarta.

*‘For me and my husband, decision-making is balanced. No one is more than the other. We just take turns as to whose opinion is better; it does not have to be my husband’s always or mine always.’* Participant in women group in urban Purworejo (FGD 8).

*‘It is already a government regulation that a household leader must be a male; de jure it is the husband, de facto it is the wife.’* Participant in men group in suburban Jakarta (FGD 10).

### ‘The environment makes the unhealthy choice the easy choice’

The information in this category reflects the environment that encourages people to eat unhealthy foods. Junk foods, i.e. fried chicken nuggets, instant noodles, etc., are widely available from street sellers, and people find eating out to be exciting. The participants acknowledged these factors as drivers for people’s excessive weight and obesity.

*‘All of my children like it (eating out), the three of them. If I take them to eat out, the oldest one eats the most. The younger one eats a lot too. He is very excited about eating out.’* Participant in men group in suburban Jakarta (FGD 10).

Inside school environments, the availability of junk food is considered to be a particularly influential factor toward children’s deteriorating nutritional status. Foods considered to be not healthy to children’s health are sold at schools; indeed, the schooling period is considered as a period when a big shift happens in children’s nutritional status and eating behaviour. The environment outside of the school environment also seems to play an important role in promoting unhealthy eating behaviour. The area surrounding a school is depicted by the informants as a place full of street sellers offering snacks, instant noodles and fried chicken nuggets to school children.

*‘I have three children. The oldest one was small before entering elementary school and did not want to eat. My brother’s child is also like that; when he/she was younger, he/she was small, and after 3rd or 4th grade of elementary school, he/she got fat. So all of them are fat now.’* Participant in women group in rural Purworejo (FGD 7).

In our interpretation, this signifies an emerging obesogenic environment, affecting the whole population and all age groups. Among adults, men seem more prone to following this pattern and lifestyle when compared with women. Within this new lifestyle there is too little time for cooking, and individuals’ eating habits are trending mainly towards the consumption of ready-made foods and noodles rather than traditional foods such as steamed rice, tempeh, vegetables, vegetable soups and eggs.

*‘My brother is overweight now because he only eats food at fast-food restaurants every day. He is already a member and he gets delivery service every day. Chicken means eating for him. One is not enough, and he has to have two pieces every day.’* Participant in women group in suburban Jakarta (FGD 12).

Nevertheless, the obesogenic environment seems to have a greater impact on children than adults. Both men and women share the same story with regard to this issue, and no differences observed in terms of area of residence; for instance, between urban and rural parts of Purworejo or between central and suburban areas in Jakarta.

*‘If I consume instant noodles as dinner for several days, I can see that it affects my weight. There is a concern, but it is not much. We are trying our best to switch to another menu, but if we are busy enough and our kids do not want to eat other meals except instant noodles, we have no choice. As long as the kids can be persuaded to eat other meals, we’ll give them other meals.’* Participant in men group in rural Purworejo (FGD 1).

### ‘Parents are concerned but not able to control children’s eating habits’

Generational relations affect parents’ abilities and approaches in dealing with their children’s eating behaviour. This came out as a common problem, as expressed by the participants. However, there are some differences between fathers and mothers in the ways that they address their children’s eating styles.

Men and women are both ‘concerned’ about children’s eating habits and are conscious of the effects of an unhealthy eating style. The problem appears in the ability of parents to control children’s eating behaviour. Despite parents’ resistance and disapproval, children have the will power to do and to eat whatever they wish, and they frequently consume junk food and sweetened beverages. According to our informants, children prefer to eat chicken nuggets, cookies and instant noodles rather than fruits, vegetables and traditional homemade foods. They maintain this behaviour by ‘crying’, ‘fighting’, ‘insisting’, ‘being upset’, ‘becoming mad’, etc. Parents, on the other hand, are incapable of preventing these habits and eventually give up.

*‘Yes, we practice good nutrition in Posyandu, such as not eating food with too many preservatives because it can cause problems with our tonsils. Sometimes we remember to practice, but most of the time we forget. Our children ask for snacks, and if we don’t buy the snacks for them, they can get upset. We don’t want them to be upset so we prefer giving them what they want.’* Participant in men group in rural Purworejo (FGD 1).

The main burden is on parents’ shoulders for not being creative enough in preparing and providing children with healthier foods and snacks and also for not being able to prevent children from buying and consuming junk food. This concern was raised by a woman in rural Purworejo:

*‘I think the point is less the creativity of parents in processing food. Children like various foods and shapes. We can mix vegetables into rice, for example. But sometimes community members are too lazy to do it. I think if parents are aware that they can make meals that are attractive to their children – it does not have to be fried Tempe or Tempe bacem (Tempe cooked with soybean sauce) all the time, but it can be processed into other menus and attractive shapes – children will be very happy. Yet it takes time and effort to raise the awareness within our community. I think that is our challenge.’* Participant in women group in rural Purworejo (FGD 3).

The other problem comes with the availability of junk food (i.e. the obesogenic environment), which makes it even more difficult for parents to control their children’s eating behaviour. Mothers seem to be more concerned and more responsible for this issue, whilst fathers comply with this obesogenic environment and would rather feed children junk food by taking them out to eat or buying take-away foods rather than cooking at home, mainly due to their lack of cooking skills. A mother expressed:

*‘When fathers take the children to go out, they tend to give children things or food that the mothers forbid. They often say to the children that it is okay to eat that food because their mother is not with them. I protested to my husband for doing that, but my husband said that it was okay for children to have that food occasionally. That makes us have different ways of giving rules to the children and it is not good for the children.’* Participant in women group in Central Jakarta (FGD 11).

There is also an unequal division of labour within households, and it seems that men have a laissez-faire approach and go with the flow whenever they are supposed to take care of children. This might also reflect the fact that the domestic responsibilities have not been shared with men, despite women’s greater involvement in paid work outside of the household.

*‘Honestly, I cannot cook. I can only cook Indomie (instant noodles) and fried rice.’* Participant in men group in suburban Jakarta (FGD 10).

## Discussion

**Figure 1.**
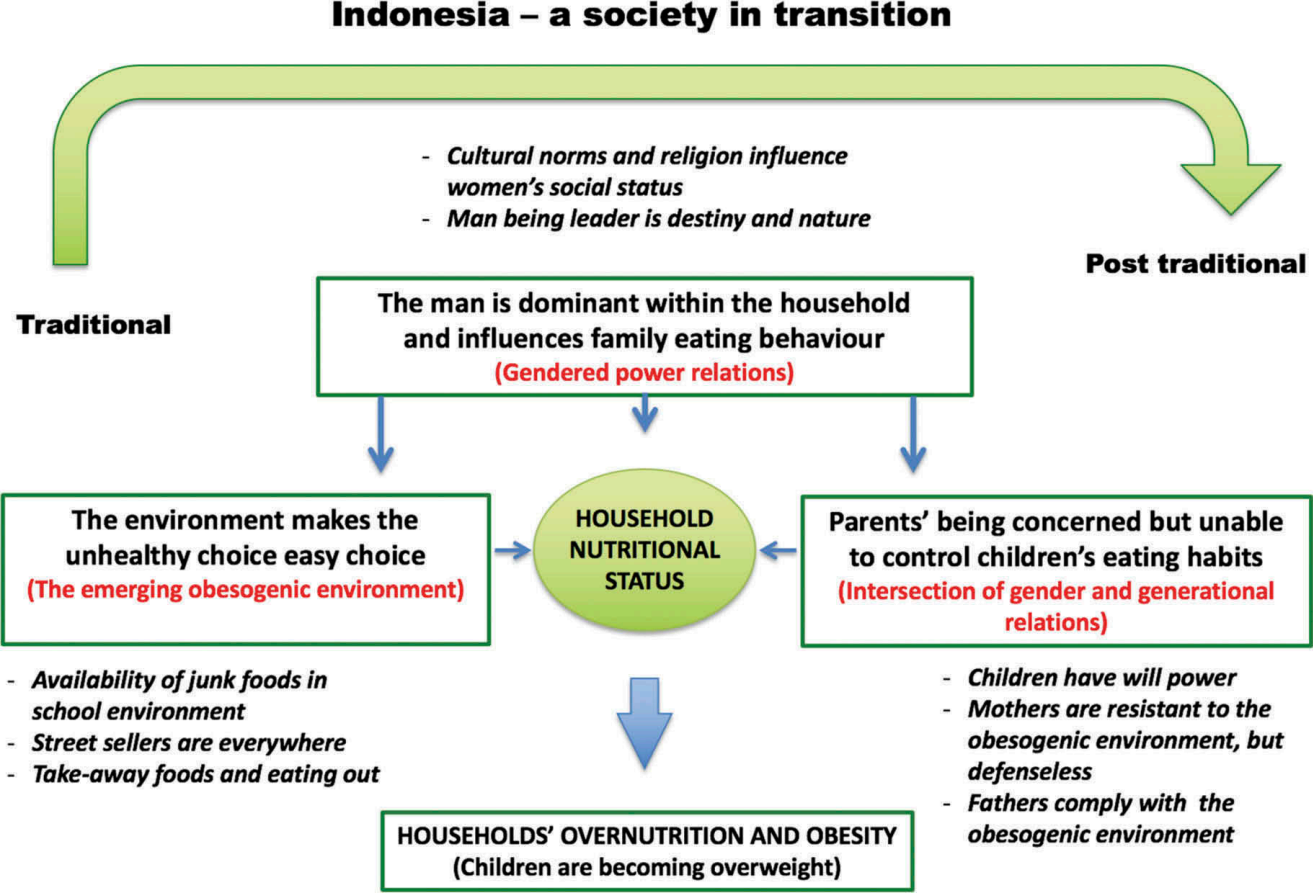
The interrelations among obesogenic environment, gendered power relations and generational relations in excess childhood weight and obesity among households

This study reveals that the parents seem to be stuck in a web of circumstances that constrain them from engaging in parenting behaviour that promotes healthy eating habits for their children. The constraints have to do with gendered power relations in the household, with generational power relations and with an environment in transition that is fuelled by a great deal of available junk foods. Our findings reveal that a trend toward unhealthy eating patterns, especially among children, and increasing excess weight among household members dominated the issue of the double burden of malnutrition that is the coexistence of overnutrition and undernutrition in a household. Based on the results from the FGDs, we constructed a model which illustrates the interrelation among the three categories: (i) the man is dominant within the family (gendered power relations), (ii) the environment making the unhealthy choice the easy choice (the emerging obesogenic environment) and (iii) parents being concerned but not able to control children’s eating habits (intersection of gender and generational relations) (Figure 1).

In the following discussion, we will use this model as point of departure to discuss the findings and how the categories are interrelated.

### Indonesia – a society in transition

We did not initially intend to study the concept of transition itself in this study, except for the nutrition transition. However, evidence of transition in all aspects of our study, including gender relations, appeared to be an inevitable single overarching factor. The high presence of street sellers and accessibility of junk food, accompanied by increasing changes in food habits, is the reality of Indonesia’s emerging obesogenic environment. This affects the nutrition of households and especially children. It is, though, important to mention that nutrition transition occurs within the larger context of economic transition, which has progressed significantly in the last few decades in Indonesia. Nonetheless, the transition among household members from being undernourished and underweight to being overweight and obese, the transition in food consumption and the gradual transition of gender roles and relations within households and society are some examples of Indonesian society undergoing change and, perhaps, becoming post-traditional. Giddens claims that ‘modernity is post-tradition’ [41]. He argues that societies cannot be completely modern if attitudes, actions or institutions are significantly influenced by a tradition that is contradictory to ‘modern reflexivity’.

### Gendered power relations

Our results show how the way households respond to this observed reality is gendered. For example, fathers in the focus groups did not consider themselves to be the main parties responsible for their children’s eating behaviour and food consumption, as it is always the mother’s ‘role’ to be the primary care giver for children. Fathers showed, however, that they had actually become part of the emerging obesogenic environment by often taking children out for fast food or by purchasing or ordering food from outside the home rather than cooking at home (in some instances, not knowing how) when mothers were not available. Mothers were concerned about and conscious of their children’s unhealthy eating patterns and strove to act against this behaviour, but they did not possess enough power against their children’s willpower.

In a society like Indonesia that has long stood at the crossroads of culture and development, there are also increasing efforts from women seeking a more gender-equal life, especially among the younger generations. Women are becoming increasingly involved in generating or earning income, contributing to the household economy and participating in community activities. This involvement in the society, which Giddens refers to as ‘modern reflexivity’, provides women with more authority. Yet, it will not enhance their position in the family hierarchy if it is reinforced with more and stronger gender sensitive laws at the institutional and national levels. Further studies are needed to investigate the effects of these changes on the health of households – particularly on the nutritional status of mothers and children – by applying the relational theory of gender.

In 1961, Geertz observed the mother and child relationship within the Javanese context as a durable and secure relationship that will last a lifetime [42]. He perceived that children respected their mothers but never addressed them in the formal style of Javanese speaking that they used when they addressed their fathers. Connell refers to this as symbolic relations, a concept that embraces the construction of gender identities and draws attention to how gender discourses are symbolised through language and gestures. Such intergenerational power relations within households might be a result of the gendered division of labour and gendered power relations between men and women. This has been reinforced by an institutionalised national ideology of gender reflected in a nationwide organisation called *Dharma Wanita* (Women’s Dedication), which considers marriage and motherhood as the final destiny for women. This hegemonic gender ideology simultaneously emphasises women’s importance and subordinates them in relation to men. Thus, women lack significant real power, both in society and in their households, despite being highly active in generating income and household financial management [25,43–45]. In fact, applying the relational theory of gender is useful for understanding the differences between the roles that women and men play in the same context, as well as their intertwining relationships of economic arrangements and power and ultimately the impact of these differences on their children’s nutritional status. Referring back to Connell’s economic, power and symbolic relations, these gendered roles and relations, values and attitudes are repeated and transmitted from one generation to the next, resulting in intergenerational power relations.

Gender equality and women’s empowerment are essential for the realisation of human rights and key to sustainable and effective development outcomes [46]. Empowerment often comes from within, as individuals empower themselves; however, societies, cultures and institutions construct conditions that strengthen or weaken possibilities for empowerment. Development models focused on income generation to achieve productivity goals should also strategically and deliberately create opportunities for women that help them attain more power at productive, reproductive and community levels [46]. Based on the results from our FGDs and existing literature, women in Indonesia have been and are indeed important income generators within the household and have access to economic resources, particularly in comparison with women from other Asian societies or Islamic cultures such as Singapore and Malaysia [25]. Yet, in order to lead household matters effectively and gain access to significant roles in society, they still confront major structural and cultural hindrances, which are strongly constrained by a patriarchal gender ideology that confines women’s independence and autonomy, especially among Javanese women [45]. Nonetheless, this situation is not unique to Javanese culture or to Indonesia, as contradictions in gender roles/ideologies/beliefs and practices have been largely recognised in many social and political settings [45]. Consequently, analytical schemes to study the structure and process of gender relations must conceptualise gender as a multidimensional experience in which norms and practices constantly interrelate.

Different methods have been proposed to investigate structures and processes in which the subordination of women occur, for example, at a given point in time [47], within a patriarchy structure [48] or within distinct societal institutions [49]. These approaches, nonetheless, dodge the flowing and dynamic social processes. As Connell points out, ‘in a real-life context, the different dimensions of gender constantly interweave and condition each other’ [50]. In fact, we have identified several links between our data and different dimensions of Connell’s theory. Our findings recognise institutionalised power relations (gender policy or ideology such as marriage law) or discursive power relations (norms and beliefs about gender based on culture or religion) as contributing to the subordination of women and dominance of men. In addition, we refer to the gendered division of labour in the form of the allocation of tasks as the production relation, as men are considered the heads of households and breadwinners while the main responsibilities of women are doing household chores and taking care of children. Such a gendered division of labour depicts particular work as masculine and other work as feminine.

The results of our study also recognise gender relations within households as subject to change rather than being a constant process. For instance, more women are becoming engaged in decision-making and earning a living, and men occasionally participate in household tasks.

### The emerging obesogenic environment

Understanding which physical/built environmental elements affect an individual’s health, as well as how best to influence them, is important for public health and urban planning. Street-vended foods, as part of an obesogenic urban environment, have become an important public health issue and a great concern to everybody in the developing world. This is mainly due to a lack of basic infrastructure for food preparation and difficulties with the large number of street food sellers [51]. The World Health Organization (WHO) has defined street foods as ‘foods and beverages prepared and/or sold by vendors in streets and other public places for immediate consumption or consumption at a later time without further processing or preparation’ [52]. In Indonesia, there are many types of street food operations, such as vendors providing fritters (mainly chicken nuggets) and energy-dense dishes, small shops (*warung*) providing snacks and beverages and small cafés (*warung makan*) providing main meals. The physical environment in most part of Indonesia has been assessed as ‘an urban environment that is fairly unfriendly to pedestrian physical activity with limited access to healthy foods’ [53]. Consequently, children and adults commuting from schools and workplaces have few options outside of their homes other than ready-made foods provided by street sellers. In their report, Shrimpton and Rokx observed a very low awareness in Indonesian society of the double burden of malnutrition and the lack of initiative in the schools for the prevention of childhood obesity. Uncovering the limited capacity of the Indonesian health system to implement nutrition interventions aimed at improving maternal and childhood nutrition, they also highlighted the lack of awareness among health professionals of both stunting and overweight/obesity as problems. Recognising this as a challenge, they suggest that there is a need to regulate the obesogenic environment (street vending) to ensure that children eat more healthily [53]. Although it is more pronounced in urban settings, we believe this situation is not unique to urban areas. The results from our FGDs confirmed the wide availability of junk foods such as fried chicken nuggets and sugary snacks for children even in rural areas.

### Intersections of gender and generational relations

Our study reveals that children constantly eat unhealthy foods, which makes them susceptible to excess weight and obesity at younger ages. This is in line with the World Bank report on the double burden of malnutrition in Indonesia, which disclosed ‘inadequate feeding practices of infants and young children which contribute to undernutrition in early childhood as well as an increased propensity for overnutrition later in the life course’ [53]. Therefore, it is essential to understand parents’ influence on their children’s eating behaviour. Children’s nutritional behaviours are, to a large extent, shaped by their family and their parents [54]. Research on the influence of parenting behaviour and its relationship with children’s nutrition behaviour showed that authoritative parenting is positively associated with children’s good eating habits, better physical activity and lower BMI z-score [55,56]. Our study, however, demonstrates that parents lack such authority in controlling children’s unhealthy eating habits.

The intersection between gender and generation stipulates a more complex and maybe a more universal picture within household relationships, in which there is an interplay of gender (parents, especially mothers) and generation (children). This intersection forms differentiated patterns of susceptibility, needs and obligations for individuals within a household. Intersectionality is defined as a theoretical and methodological tool for analysing how historically specific kinds of power differentials and/or constraining normativities, based on discursively, institutionally and/or structurally constructed sociocultural categories such as gender, ethnicity, race, class, sexuality, age/generation, dis/ability, nationality and mother tongue interact and produce different kinds of societal inequalities and unjust social relations [57]. A fundamental assumption in intersectional theory is that the intersectional identities are emergent and defined in relation to one another [58,59]. The intersections of gender and generation were not what we initially intended to study. However, the analyses resulted in identifying generation as a significant power dimension clearly intersecting with gender. The power structure of intersection between gender (parents, especially mothers being at the end of the family hierarchy, ‘taking the second turn’) and generation (children, ‘put first’ of both mothers and fathers) might provide an understanding of why mothers are incapable of controlling their children’s eating behaviour.

### Methodological considerations

To increase trustworthiness and the credibility of our study, we used triangulation of researchers, peer debriefing and prolonged engagement – techniques described by Lincoln and Guba [60] and Dahlgren et al. [35]. Firstly, triangulation of researchers was evident as the research team consisted of four researchers with multidisciplinary backgrounds in nutrition, public health and gender studies. These different areas of expertise were used when planning for the data collection and interview guide, during the process of analysis and in the final drafting of the results, in which we mirrored the emerging categories with theories of gender, intersectionality, food and health. The result presented here is a negotiated outcome from these triangulating processes. Secondly, a member of the research team (MH) had prolonged engagement with and deep knowledge of Javanese society. In addition, the field assistant was Javanese and had adequate experience in fieldwork and qualitative studies. This helped in collecting and analysing data with cultural sensitivity. Thirdly, we have held peer-debriefing sessions in order to get input into the different steps of analysis.

It might be argued that our results gave an overdue importance to just the three proximal categories identified in the FGDs. We acknowledge that underlying causes of malnutrition are indeed multifaceted; however, in this study, we tried to reflect what is important from the participants’ point of view rather than applying our pre-understanding of the phenomena during the FGDs. We were not able to fully control the process of selecting informants as we had to rely on community leaders to select and invite household members of different socio-economic backgrounds from different parts of the village. In some of the focus groups, the majority of the participants were from the same association (i.e. village hall) or neighbourhood, which might have contributed to their willingness to share their thoughts and experiences openly. However, it is very unlikely that our findings project the views of just one group of individuals as we conducted several FGDs in different settings, among many different men and women with different backgrounds. Another limitation of the study was that the first author (MV) did not have a prolonged engagement with Javanese society and does not speak the language and, therefore, did not moderate the FGDs. In that sense, we had to rely on the trained field assistant and her capability of steering and moderating the discussions. She was, however, fully informed and aware of the purpose of the study and took an active role in the summary discussions directly after each FGD session. Despite this drawback, we regard the combination of an ‘insider’s perspective’ (means of access, cultural sensitivity and understanding) and an ‘outsider’s perspective’ in the research team (possibility of detecting patterns that may be hidden by the blinder of familiarity) as a productive factor for the different steps of analysis.

Qualitative studies do not try to make statistical generalisations, as they often deal with too small a number of participants to study a phenomenon deeply and comprehensively. Instead, the sample selection is carried out to achieve an analytical generalisation; therefore, the participants are selected to contribute to the theory that is being developed [35]; hence the knowledge we gained from this study might be transferable beyond the study sample to other similar social contexts from a theoretical point of view.

## Conclusion

We did extensive fieldwork in Central Java and Jakarta to explore and understand what contributes to the double burden of malnutrition within a household in the Indonesian context. The growing problem of overnutrition and obesity, especially among children, overshadowed the issue of the double burden of malnutrition. Obviously, there is no single solution that can address the transition of malnutrition within households; however, we found women’s low social status as a result of gendered power relations with a generational dimension, coupled with an emerging obesogenic environment filled with lots of junk food and unhealthy eating habits, to be the main components of the nutrition transition.

We are aware of the fact that gender equality and women’s empowerment can only materialise if the context allows. Nevertheless, community health and nutrition programmes should help both women and men within the context of households to acknowledge and respect women’s status. More importantly, they should also hold men accountable for children’s nutritional habits and consider them as an important quality of an ideal goal for gender equality. Furthermore, it is imperative to recognise the implication of the accessibility and availability of junk food, especially among children in Indonesia. The circumstances in which people live are often beyond their control. Therefore, encouraging and supporting the production of healthy street foods and the inclusion of educational materials for food and nutrition in the school curriculum could be some examples of how to fight against this emerging obesogenic environment. Moreover, in order to prevent children consuming junk food outside of the home, community programmes should also educate parents about healthy lifestyles and encourage them to prepare more appealing and attractive healthy, nutrient-rich foods for children.

## Paper context

This study reveals that the parents seem to be stuck in a web of circumstances that constrain them from engaging in parenting behaviour that promotes healthy eating habits for their children. The constraints have to do with gendered power relations in the household, with generational power relations and with an environment in transition that is fuelled by a great deal of available unhealthy foods.
